# Crambescin C1 Acts as A Possible Substrate of iNOS and eNOS Increasing Nitric Oxide Production and Inducing *In Vivo* Hypotensive Effect

**DOI:** 10.3389/fphar.2021.694639

**Published:** 2021-07-07

**Authors:** Juan A. Rubiolo, Emilio Lence, Concepción González-Bello, María Roel, José Gil-Longo, Manuel Campos-Toimil, Eva Ternon, Olivier P. Thomas, Antonio González-Cantalapiedra, Henar López-Alonso, Mercedes R. Vieytes, Luis M. Botana

**Affiliations:** ^1^ Departamento de Zoología, Genética y Antropología Física, Universidad de Santiago de Compostela, Lugo, Spain; ^2^ Facultad de Ciencias Bioquímicas y Farmacéuticas–Ministerio de Ciencia, Centro Científico y Tecnológico Acuario del Río Paraná, Tecnología e Innovación Productiva de Santa Fe, Universidad Nacional de Rosario, Rosario, Argentina; ^3^ Centro Singular de Investigación en Química Biolóxica e Materiais Moleculares (CiQUS) and Departamento de Química Orgánica, Universidade de Santiago de Compostela, Santiago de Compostela, Spain; ^4^ Departamento de Farmacología, Facultad de Veterinaria, Universidad de Santiago de Compostela, Lugo, Spain; ^5^ Departamento de Farmacología, Facultad de Farmacia, Farmacia y Tecnología Farmacéutica, Universidad de Santiago de Compostela, Santiago de Compostela, Spain; ^6^ Fisiología y Farmacología de las Enfermedades Crónicas (FIFAEC), CIMUS, Universidad de Santiago de Compostela, Santiago de Compostela, Spain; ^7^ CNRS, OCA, IRD, Géoazur, Université Côte d’Azur, Valbonne, France; ^8^ Marine Biodiscovery, School of Chemistry and Ryan Institute, National University of Ireland Galway, Galway, Ireland; ^9^ Departamento de Anatomía, Producción Animal y Ciencias Clínicas Veterinarias, Hospital Veterinario Universitario Rof Codina, Facultad de Veterinaria, Universidad de Santiago de Compostel, Lugo, Spain; ^10^ Departamento de Fisiología, Facultad de Veterinaria, Universidad de Santiago de Compostela, Lugo, Spain

**Keywords:** crambescin, nitric oxide synthase, docking, molecular dynamics simulations, hypotension, metallothionein, HepG2 cells

## Abstract

Crambescins are guanidine alkaloids from the sponge *Crambe crambe*. Crambescin C1 (CC) induces metallothionein genes and nitric oxide (NO) is one of the triggers. We studied and compared the *in vitro*, *in vivo, and in silico* effects of some crambescine A and C analogs. HepG2 gene expression was analyzed using microarrays. Vasodilation was studied in rat aortic rings. *In vivo* hypotensive effect was directly measured in anesthetized rats. The targets of crambescines were studied *in silico*. CC and homo-crambescine C1 (HCC), but not crambescine A1 (CA), induced metallothioneins transcripts. CC increased NO production in HepG2 cells. In isolated rat aortic rings, CC and HCC induced an endothelium-dependent relaxation related to eNOS activation and an endothelium-independent relaxation related to iNOS activation, hence both compounds increase NO and reduce vascular tone. *In silico* analysis also points to eNOS and iNOS as targets of Crambescin C1 and source of NO increment. CC effect is mediated through crambescin binding to the active site of eNOS and iNOS. CC docking studies in iNOS and eNOS active site revealed hydrogen bonding of the hydroxylated chain with residues Glu377 and Glu361, involved in the substrate recognition, and explains its higher binding affinity than CA. The later interaction and the extra polar contacts with its pyrimidine moiety, absent in the endogenous substrate, explain its role as exogenous substrate of NOSs and NO production. Our results suggest that CC serve as a basis to develop new useful drugs when bioavailability of NO is perturbed.

## Introduction

**FIGURE 1:**
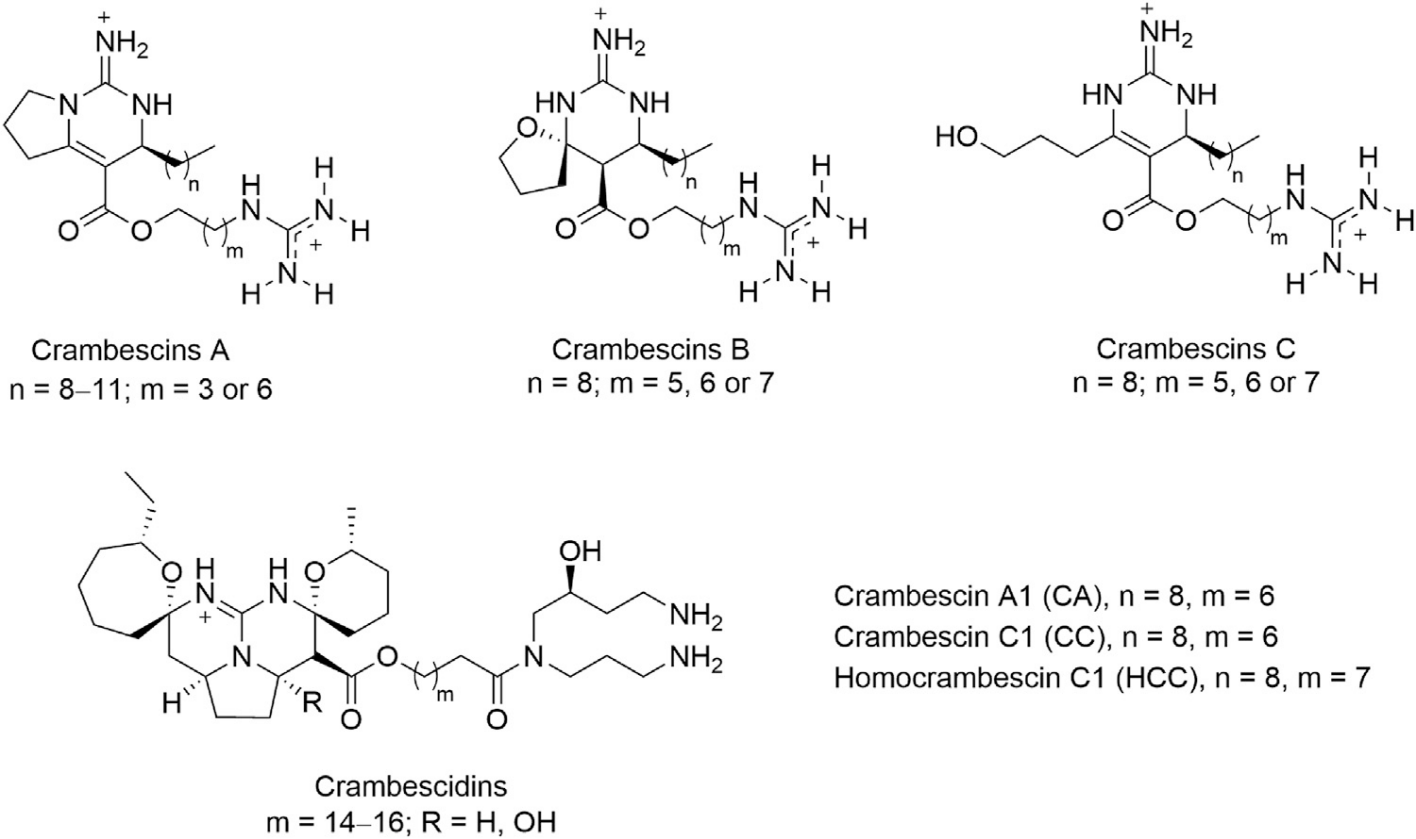
General chemical structures of guanidine alkaloids from the marine sponge *crambe crambe,* crambescins (A–C) and crambescidins, and compounds studied in this work, CA, CC, and HCC.

Some sponge species of the order Poecilosclerida are known to produce a diverse array of unique bioactive polycyclic guanidine alkaloids (Berlinck et al., 2010). A sponge of this order, *Crambe crambe*, is largely distributed in shallow waters of the Mediterranean sea and the Macaronesian archipelagos. The natural products present in this sponge have been extensively studied during the last decades that led to the description of two families of polycyclic guanidine alkaloids (PGA), the crambescins and crambescidines (Bondu et al., 2012). A wide diversity of molecular variations of these two type of compounds has been described and the activity of some have been studied by us and others (Aoki et al., 2004; Olszewski et al., 2004; Lazaro et al., 2006; Suna et al., 2007; Martin et al., 2013; Rubiolo et al., 2013; Rubiolo et al., 2014; Roel et al., 2015). In particular, crambescin C1 (CC, Figure 1) was reported as a potent inducer of metallothioneins (MTs) in cell cultures at non-toxic concentrations (Roel et al., 2015).

MTs are cysteine-rich and low molecular weight proteins (61–62 amino acids) able to coordinate a variety of metal ions through their cysteine residues (Haq et al., 2003). Four isoforms of MT (MT1, MT2, MT3, and MT4) have been identified. MT1 and MT2 are expressed ubiquitously in all mammalian cells (Miles et al., 2000), while MT3 is produced mainly in brain (Palmiter et al., 1992), and MT4 expression is restricted to skin epithelial cells and tongue (Quaife et al., 1994). Even though the physiological function of these proteins still remains unclear, they have proved to be involved in heavy metals detoxification, homeostatic regulation of essential metals, as well as cell protection against oxidative injury (Miles et al., 2000). MTs were also reported to be induced by nitric oxide (Schwarz et al., 1995) and to have a role in NO signaling (Pearce et al., 2000). In this regard, MTF-1, the central regulator of MT-1 and MT-2 gene expression, is induced by increased zinc or heavy metal ions (e.g., cadmium) concentration, hypoxia, oxidative stress, stress hormones (glucocorticoids), NO, and temperature (Dalton et al., 1994; Murphy et al., 1999; Gunther et al., 2012).

To better understand the MT-inducing effect of PGA we used crambescin C1 (CC) as a model compound, and we first explored the transcriptomic changes in HepG2 cells induced by CC, but also by two structurally similar crambescins, homocrambescin C1 (HCC) and crambescin A1 (CA) (Figure 1). Specifically, HCC has an additional methylene group in the side chain containing the guanidinium group, and CA is a cyclic derivative of CC lacking the terminal hydroxyl group. We hypothesized that MT induction could be mediated by oxidative stress and/or NO. To confirm or rule out this hypothesis we assayed oxidative stress induction and NO production *in vitro*. We then confirmed the NO production *in vivo*, and finally, the molecular basis of the increase of NO production mediated by iNOS and/or eNOS was studied through molecular docking and Molecular Dynamics (MD) simulation studies.

## Materials and Methods

### Crambescins Isolation and Purification

Crambescin C1 (CC), crambescin A1 (CA), and homocrambescin C1 (HCC) were purified from the sponge *Crambe crambe* as described previously (Bondu et al., 2012). The purity of the tested compounds analyzed by HPLC-MS and NMR, was of at least 95%. Compounds were tested as solutions in DMSO. For *in vitro*, and *in vivo* assays, the final DMSO concentration of these compounds was always ≤0.5% and in control experiments we used the highest DMSO concentration to rule out any solvent interference in the results observed.

### HepG2 Cell Culture

HepG2 cells were cultured in E-MEM medium (eagle’s minimum essential medium, SIGMA) supplemented with 10% FBS (fetal bovine serum, Cambrex), 100 UI ml^−1^ penicillin (Roche) and 1.2 mM streptomycin (Roche) at 37°C in a humidified 5% CO_2_ atmosphere. Cells were cultured in 6 cm diameter plates and in 96 well plates for the different experiments performed in this work.

### DAF-2-DA Assay for NO Detection

HepG2 cells cultured in 96 well plates until confluent were exposed to 10 mM DAF-2-DA (4,5-diaminofluorescein diacetate) dissolved in fresh culture medium for 1 h. After treatment, the medium was discarded and replaced with fresh medium supplemented with CC, CA, HCC (5–10 µM) or vehicle in the case of controls with a replicate number of five for each treatment. Cells were incubated in the presence of the toxins for 30 and 60 min. Finally, fluorescence was determined at 485 nm (excitation) and 515 nm (emission) with a Bio-Tek Sinergy plate reader. One way ANOVA was used to determine significant differences between treatments followed by a Tukey post hoc test. Differences between treatments were considered significant when *p* < 0,05 (*n* = 5).

### Reactivity Studies in Rat Aortic Rings

Experiments (within officially authorized protocols) were conducted on male Wistar rats aged 4–6 months (320–380 g). The rats were killed by CO_2_ inhalation and exsanguination. The thoracic aorta was rapidly removed and placed in a Petri dish with Krebs bicarbonate solution (KBS; composition mM: NaCl, 119; CaCl_**2**_^**.**^2H_**2**_O, 1.5; NaHCO_**3**_, 25; KCl, 4.7; MgSO_**4**_^**.**^7H_**2**_O, 1.2; KH_**2**_PO_**4**_, 1.2; glucose, 11; dexamethasone 0.001; pH 7.4; 37°C), oxygenated with carbogen (95% O_**2**_+5% CO_**2**_), cleaned of perivascular fat and cut into 2–3 mm long cylindrical rings. In some rings, the endothelium was removed (rubbed rings) by gently rubbing the intimal surface with a cotton thread.

The aortic rings were immediately transferred to an organ bath containing 2 ml of KBS thermoregulated at 37°C and bubbled with carbogen. Two stainless steel pins were inserted through the lumen of each arterial segment: one pin was fixed to the organ bath and the other was connected to a force-displacement transducer to record the isometric tension using a calibrated computerized system. The preparations were then equilibrated at a resting tension of 4.5 g for at least 1 h, replacing KBS every 15 min. Afterwards, a contraction was induced by the addition of L-phenylephrine hydrochloride (phenylephrine, 1 µM). Once the contraction stabilized, a single concentration of acetylcholine chloride (1 µM) was added to the bath to assess the endothelial integrity of the preparations (see supplementary Figure S1). After assessing the presence of a functional endothelium, vascular tissues were allowed to recuperate for at least 1 h, replacing KBS every 15 min. Then (see supplementary Figure S1), intact and rubbed aortic rings were contracted again with a single concentration of phenylephrine (1 µM). After stabilizing the contractions, CC and HCC were added to the bath in progressively increasing cumulative concentrations (0.1–10 μM). After the highest concentration of crambescins was tested, the rings were challenged with N^G^-nitro-L-arginine (L-NA; 30 μM). Then the preparations were washed for 1 h and the response to phenylephrine was evaluated again in order to assess their functionality. In a subset of experiments carried out in a KBS without dexamethasone, rubbed rings were incubated with lipopolysaccharide (*E. coli* type, Serotype 055: B5; LPS; 10 μg/ml; 5 h) in order to induce the expression of iNOS previously to add phenylephrine and the crambescins; in these experiments, after the highest concentration of crambescins was tested, the rings were challenged with *N*-([3-(aminomethyl)phenyl]methyl)ethanimidamide dihydrochloride **(**1,400 w; 10 μM).

### Microarray Assay and Analysis

HepG2 cells cultured in 6 cm plates were treated with 10 mM CC, CA and HCC for 24 h. Total RNA was then extracted using the Aurum^TM^ Total RNA Mini Kit (Biorad) following the manufacturer instructions. Concentration and integrity of extracted RNA were determined with a NanoDrop 2,000 (Thermo Scientific) and a Bioanalyzer 2,100, using the RNA6000 nanoreagents kit (Agilent), respectively. Complimentary DNA was synthesized using the cDNA Synthesis System (Roche) and then cleaned up with a High Pure PCR Purification Kit (Roche). The cDNA was labeled using the NimbleGen One Color DNA Labeling Kit (Roche). The labeled cDNA from each sample (5 µg) was hybridized onto NimbleGen microarrays (100718_HG18_opt_expr_HX12; Roche) using the specific kit NimbleGen Hybridization Kit (Roche) in a NimbleGen HS4 mixer (Roche). After hybridization, microarrays were washed using the NimbleGen Wash Buffer Kit (Roche) and scanned with a NimbleGen MS200 scanner (Roche).

The DEVA 1.2.1 software was used to extract and segment the scanned images (Roche) and to perform the normalization of the fluorescence intensities using Robust Microarray Averaging (RMA). Normalized data was used to determine differential expression between conditions and assayed molecule using the TM4 Microarray Software Suite (Saeed et al., 2003; Supplementary Table S1 contains the normalized intensity with error, one transcript per line, used for the analysis presented in this work). Clustering was performed using K-means clustering (KMC). Enrichment analysis was performed using the R library ReactomePA (Yu and He, 2016) which implements gene set enrichment analysis based on the REACTOME (https://reactome.org/) database. A *p* value cut off of 0,05 with Benjamini-Hochberg correction for the false discovery rate was selected to identify significantly altered metabolic pathways, and plotting was done using Matplotlib (Hunter, 2007).

### Molecular Studies

These studies were carried out using the program GOLD (Verdonk et al., 2003) version 5.2 and the geometries found in the available crystal structures of eNOS/Heme/BH4/L-Arg (PDB code 4D1O, 1.82 Å (Li et al., 2014)) and iNOS/Heme/BH4/L-Arg (PDB code 1NSI, 2.55 Å (Li et al., 1999)) enzyme complexes. Ligand geometries were minimized using the AM1 Hamiltonian as implemented in the program Gaussian 09 (Frisch et al., 2009) and used as MOL2 files. Each ligand was docked in 25 independent genetic algorithm (GA) runs, and for each of these a maximum number of 100000 GA operations were performed on a single population of 50 individuals. Operator weights for crossover, mutation and migration in the entry box were used as default parameters (95, 95, and 10, respectively), as well as the hydrogen bonding (4.0 Å) and van der Waals (2.5 Å) parameters. The dimer was used in these studies. The position of L-Arg in both structures was used to define the active site and the radius was set to 10 Å. The “flip ring corners” flag was switched on, while all the other flags were off. The GOLD scoring function was used to rank the ligands by fitness.

### Molecular Dynamics Simulation Studies

1) *Ligand minimization.* The ligand geometries of the highest score solution obtained by docking were minimized using a restricted Hartree–Fock (RHF) method and a 6–31G(d) basis set, as implemented in the ab initio program Gaussian 09 (Frisch et al., 2009). Partial charges were derived by quantum mechanical calculations using Gaussian 09, as implemented in the R.E.D. Server (version 3.0), (Frisch et al., 2009; Vanquelef et al., 2011), according to the RESP (Dupradeau et al., 2010) model. Ligands were manually docked into the active site as it was obtained by docking. The missing bonded and non-bonded parameters were assigned, by analogy or through interpolation, from those already present in the AMBER (Case et al., 2005) database (GAFF) (Wang et al., 2004; Wang et al., 2006).
2) *Generation and minimization of the complexes.* Simulations of the NOS/Heme/BH4/L complexes (L = CC, CA) were carried out using the enzyme geometries in PDB codes 4D1O and 1NSI obtained by docking. These studies were also carried out with the natural substrate (Arg) as a control. For the eNOS enzyme, the coordinates of the unsolved residues 106–119 were modeled using the web-based ModLoop server (Fiser and Sali, 2003). Computation of the protonation state of titratable groups at pH 7.0 was carried out using the H^++^ Web server (Gordon et al., 2005). Addition of hydrogen and molecular mechanics parameters from the ff14SB (Maier et al., 2015) and GAFF force fields, respectively, were assigned to the protein and the ligands using the LEaP module of AMBER Tools 15 (Wang et al., 2004; Case et al., 2005; Wang et al., 2006; Case et al., 2015). As a result of this analysis, His371 was protonated in δ position. Heme parameters and Zn^2+^ parameters used with the AMBER force field were included (Clinic,; Manchester,). The protein was immersed in a truncated octahedron of ∼25,000 TIP3P water molecules and neutralized by addition of sodium ions (Jorgensen et al., 1983; Aqvist, 1990; Case et al., 2005). For the eNOS complex, the system was minimized in five stages: 1) minimization of the loop involving residues 106–119 (1,000 steps, first half using steepest descent and the rest using conjugate gradient); 2) minimization of the ligands, Zn^2+^ ions and metal bonded cysteine residues [For eNOS: Cys94, Cys99 and Cys184; For iNOS, Cys110, Cys115 and Cys200] (1,000 steps, first half using steepest descent and the rest using conjugate gradient); 3) minimization of the solvent and ions (5,000 steps, first half using steepest descent and the rest using conjugate gradient); 4) minimization of the side chains, waters and ions (5,000 steps, first half using steepest descent and the rest using conjugate gradient); 5) final minimization of the whole system (5,000 steps, first half using steepest descent and the rest using conjugate gradient). A positional restraint force of 50 kcal mol^−1^ Å^−2^ was applied to the whole protein during the stages a–b and to *a* carbons during the stages c–d, respectively. For the iNOS complex, the system was minimized in only four stages (b–e).
3) *Simulations.* MD simulations were performed using the pmemd. cuda_SPFP (Goetz et al., 2012; Le Grand et al., 2013; Salomon-Ferrer et al., 2013) module from the AMBER 14 suite of programs. Periodic boundary conditions were applied, and electrostatic interactions were treated using the smooth particle mesh Ewald method (PME) (Darden et al., 1993) with a grid spacing of 1 Å. The cutoff distance for the non-bonded interactions was 9 Å. The SHAKE algorithm (Ryckaert et al., 1977) was applied to all bonds containing hydrogen using a tolerance of 10^–5^ Å and an integration step of 2.0 fs. The minimized system was then heated at 300 K at 1 atm by increasing the temperature from 0 to 300 K over 100 ps and by keeping the system at 300 K another 100 ps. A positional restraint force of 50 kcal mol^−1^ Å^−2^ was applied to all *a* carbons during the heating stage. Finally, an equilibration of the system at constant volume (200 ps with positional restraints of 5 kcal mol^−1^ Å^−2^ to *a* carbons) and constant pressure (another 100 ps with positional restraints of 5 kcal mol^−1^ Å^−2^ to *a* carbons) were performed. The positional restraints were gradually reduced from 5 to 1 mol^−1^ Å^−2^ (5 steps, 100 ps each), and the resulting systems were allowed to equilibrate further (100 ps). Unrestrained MD simulations were carried out for 150 ns. System coordinates were collected every 10 ps for further analysis. Figures depicting structures were prepared using PYMOL (DeLano, 2008). The cpptraj module in AMBER 14 was used to analyze the trajectories and to calculate the root-mean-square deviations (RMSD) of the protein during the simulation (Roe and Cheatham, 2013).

### Binding Free Energies Calculations

The binding free energy for CC, CA, and Arg was calculated by the MM/PBSA (Miller et al., 2012) approach implemented in Amber Tools 1.5. ante-MMPBSA. py module was used to create topology files for the complex, enzyme and ligands and binding free energies were calculated with the MMPBSA. py module. A single trajectory approach was used to calculate binding free energies considering only the last 100 ns (401 snapshots) of the 150 ns MD trajectories (NOS/Heme/BH4/L) (L = CC, CA, Arg). The Poisson-Boltzmann (PB) and Generalized Born (GB) implicit solvation models were employed. The latter model provided relative free energy values more in agreement with the experimental results.

### *In vivo* Experiments

Experimental protocols were approved by the Ethical Committee of the University of Santiago de Compostela (06/19/LU-002). Sprague–Dawley female rats aged 8–16 weeks (180–260 g) were used to assay hypotension induced by CC. The rats were housed in a temperature- and humidity-controlled room (21 ± 2°C, 50 ± 5% relative humidity) and maintained on a 12 h/12 h light/dark cycle. They were caged with free access to food and water. For the experimental procedures, the rats were anesthetized with isoflurane (FI ISO 1.52%), one catheter was placed in the left jugular vein for compound administration (0,1 ml of physiological solution plus vehicle or 10 mM CC), and arterial blood pressure (ABP) was measured by placing a catheter in the right carotid artery. After injection and basal tension recovery, electrocardiography was recorded and arterial blood pressure was measured every 5 min using a direct method with a Mindray iPM12 VET Multi-parameter Monitor (Shenzhen, China) during 20 min. Three independent measurements of ABP were done for each rat. Data are reported as systolic ABP (SAP), diastolic ABP (DAP), and mean ABP (MAP).

### Data and Statistical Analysis

The data and statistical analysis comply with the recommendations on experimental design and analysis in pharmacology (Curtis et al., 2018).

All the results are expressed as means ± the standard error of the mean (SEM); ‘*n*’ indicates the number of observations, one in each animal. In rat aortic rings experiments, a one-way analysis of variance was used for comparisons among the different experimental data groups; Bonferroni test was used to make specific comparisons if significant differences among groups were found. The *p* < 0.05 was established to consider statistically significant results. The contraction produced by phenylephrine was used to calculate the percent of response of aortic rings to CC and HCC.

## Results

### Effect of Crambescins on the Transcriptomic Profile of HepG2 Cells

**FIGURE 2:**
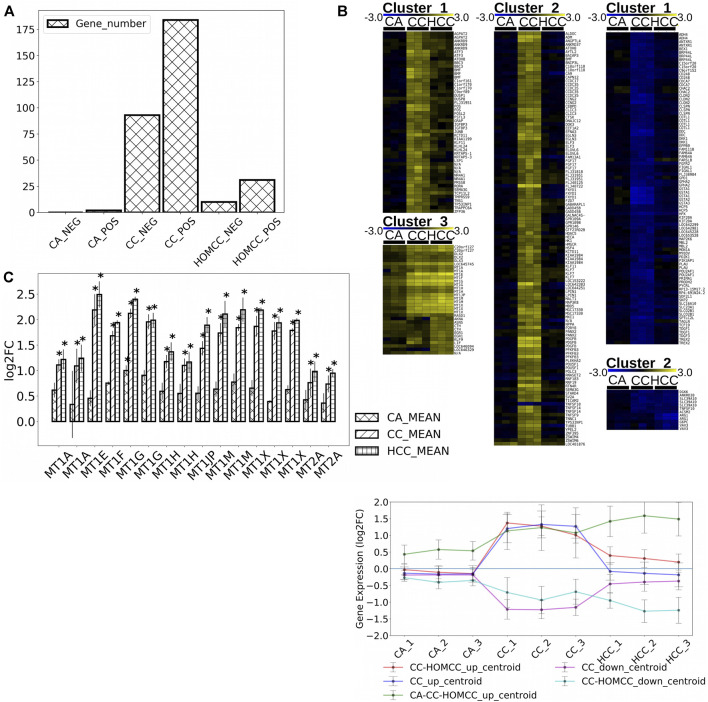
**(A)** Number of genes significantly altered (*p* < 0,05) by 10 μM CC, CA, and HCC with a logarithmic fold change higher to 1 or lower to –1. **(B)** Gene clusters obtained for up-regulated (cluster 1: up-regulated by CC and HCC, cluster 2: up-regulated by CC, and cluster 3: up-regulated by CC, HCC, and CA) and down-regulated (cluster 1: down-rebulated by CC, down-regulated by CC, and HCC) genes in HepG2 cells treated with CC, CA, and HCC shown as heat maps and as a plot of the mean expression of all genes shown as centroids of combined expression for each cluster with errors, representing the standard deviation of expression within each cluster. **(C)** Metallothionein gene expression in response to treatments with 10 μM CC, CA, and HCC. The plot shows the mean expression variation for each MT respect to controls (*p < 0.05, *n* = 3).

**FIGURE 3:**
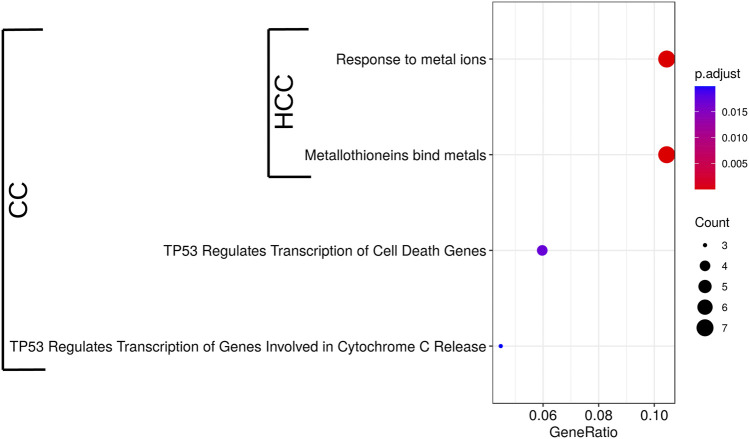
Significant Reactome PA pathways up-regulated by 10 µM CC and HCC in HepG2 cells after 24 h. Induced pathways identified in the microarrays analysis were used to perform a pathway enrichment analysis. Pathways significantly enriched are shown (*p* < 0.05, *n* = 3). The circle diameter is proportional to the number of induced genes detected for each pathway, and the gene ratio is the proportion of genes in the whole pathway induced by the treatment.

We previously showed that CC induced the expression of MTs in HepG2 cells increasing cell survival in the presence of oxidative stress (Rubiolo et al., 2014). To better understand the mode of action of crambescins, the transcriptomic changes induced by CC were compared with those induced by two members of this family of compounds, HCC and CA. To this end, the most differentially expressed genes (log2fold_change >|1| with *p* < 0.05) in HepG2 cells after treatment of each compound (10 µM for 24 h), were selected. This resulted in 184, 31, and two genes induced and 93, 2, 0 genes repressed for CC, HCC, and CA, respectively (Figure 2A; Supplementary Table S1). These genes were then clustered to determine differential expression profiles between the different compounds. As a result, three clusters of induced genes were obtained, specifically: 1) one comprised genes induced by CC and HCC but not by CA; 2) a second one with the highest number of genes, that included those solely induced by CC; and 3) a third cluster that included genes induced by the three compounds tested (Figure 2B). Repressed genes produced two clusters: 1) a first one with the highest number of genes only repressed by CC; and 2) a second one composed of genes repressed by both CC and HCC (Figure 2B). Importantly, the cluster that encompasses genes induced by the three compounds studied also included all metallothionein genes. These genes were significantly induced by CC and HCC, while a much lower effect was observed for CA (Figure 2C). Reactome PA pathway analysis for the induced genes by each compound identified four pathways induced by CC, while only two appeared induced by HCC (Figure 3). As expected, no pathway induced only by CA was detected. No pathway appeared significantly repressed by the selected genes for the three compounds after Reactome PA analysis. Induced pathways clearly pointed to an effect on MTs for CC and HCC. The remaining two induced pathways are involved in apoptosis which, as we have previously reported, is mildly observed in 10 µM CC treated HepG2 cells. Since CC showed the highest effect among the guanidine compounds tested, this derivative was selected for the subsequent studies. However, for comparative purposes, CA and HCC were also included in selected experiments.

### *In vitro* Assessment of NO Production in Response to Crambescins

**FIGURE 4:**
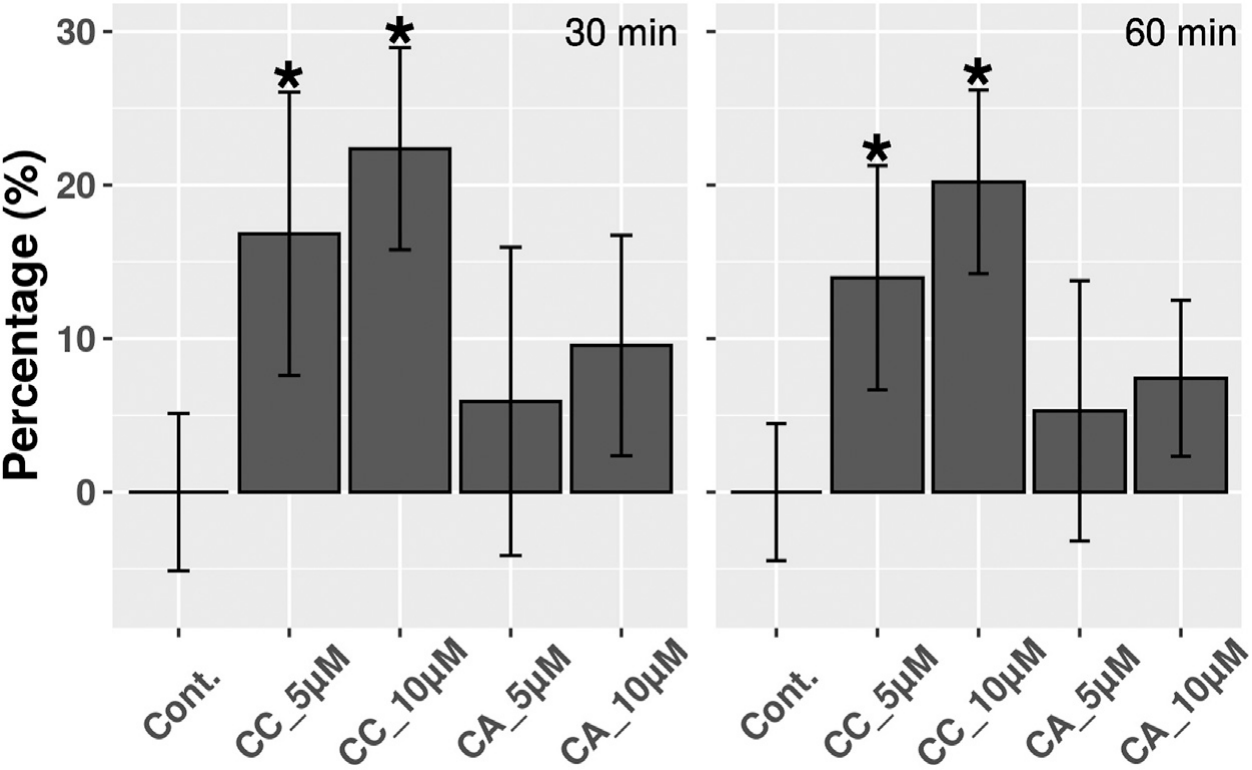
NO detection in HepG2 cells treated with 5 and 10 µM CC and CA during 30 and 60 min. Results are expressed as percentages and differences were considered significant (*) when *p* < 0.01 (*n* = 3).

There are several inducers of MT expression including oxidative stress and NO. Since we previously observed that the induction of MTs by CC was cytoprotective against reactive oxygen species (Rubiolo et al., 2014), a preconditioning effect by CC through the induction of non-lethal oxidative stress by itself, which in turn would induce MTs rendering cells with higher tolerance to this type of stress, could be behind this fact. To evaluate this hypothesis, the production of reactive oxygen species in HepG2 cells cultures after treatment with CC was analyzed. However, no increase in reactive oxygen species was detected (results not shown). Alternatively, the NO production in the same type of cultures after treating them with the most and the less active compounds, CC and CA, respectively, was evaluated. In this case, for cells treated with CC, a dose dependent increase in NO production was identified, showing increases between 15—20% after 30 with no further change after 60 min. On the contrary, for cells treated with CA, no increase in NO production was observed (Figure 4).

**FIGURE 5:**
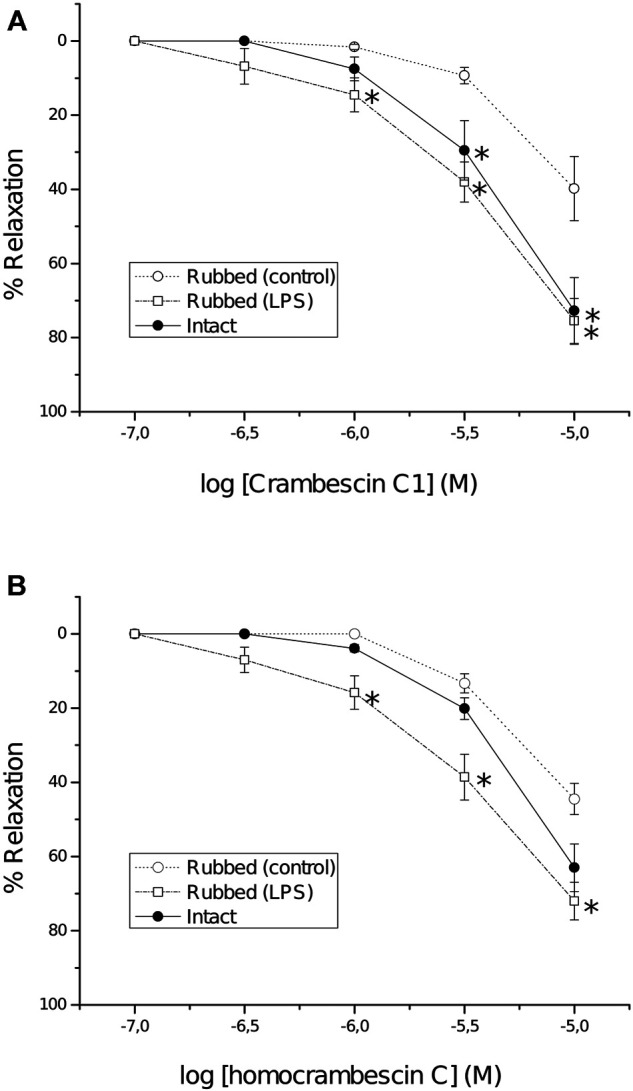
Concentration-response curves of CC **(A)** and HCC **(B)** in intact (full symbols) and rubbed (open symbols) rings of thoracic aorta pre-contracted with phenylephrine (1 µM). Before obtaining the concentration response curves, some rubbed rings were incubated with lipopolysaccharide (10 μg/ml) for 5 h (LPS). Each point represents the mean response of at least four aortic rings; S.E.M. is represented by vertical lines. *p < 0.05 vs. control rubbed rings.

Moreover, considering that NO is a potent vasodilator, the possible relaxant effect of CC and HCC was determined *in vitro,* using rat aortic rings. It is worth highlighting that CA was not included in this assay due to its lack of NO production. CC and HCC induced a dose dependent relaxation of phenylephrine pre-contracted intact and rubbed aortic rings, these last in the absence (control) or in the presence (5 h) of lipopolysaccharide (LPS, iNOS inducer; rubbed-LPS rings). When compared to control rubbed rings, a significant higher relaxation was observed in intact as well as in rubbed-LPS rings. CC and HCC effect started at 3 µM in intact rings and at 1 µM in LPS-rubbed rings; at 10 μM, the relaxant effect in both groups of rings was quite important, approximately a 70% (Figure 5). Treatment of the intact rings with N^G^-nitro-L-arginine (L-NA; eNOS inhibitor), almost completely reverted the relaxing effect of CC and HCC (Supplementary Figure S1). In the same way, *N*-([3-(aminomethyl)phenyl]methyl)ethanimidamide dihydrochloride (1400W; iNOS inhibitor) nearly completely reverted the relaxing effect of CC and HCC in rubbed-LPS rings. Regarding the relaxant effect of CC and HCC in rubbed rings, while it is small at low concentrations of the drugs, it reaches approximately a 50% at the highest concentration used, 10 µM. Neither L-NA nor 1400 W, reverted the relaxant effect of CC and HCC in rubbed rings.

### *In silico* Molecular Analysis

To get an insight on the molecular basis of the potentiating effect of CC on the NOS activity, the interaction in atomic detail of CC on the active site of iNOS and eNOS enzymes was first studied by molecular docking using the program GOLD and the enzyme coordinates found in the crystal structures of eNOS and iNOS in complex with the natural substrate and cofactors, i.e., L-arginine, 5,6,7,8-tetrahydrobiopterin (BH4) and heme group. In this study CA was also included, to get a better understanding of the reasons at molecular level behind the opposite observed effects, thus, while CC induced high NO production and vasodilation, NO production for CA was not identified. The proposed binding modes were further analyzed by MD simulation studies to assess the stability and therefore the reliability of the postulated binding. This was performed with the highest score solutions obtained by docking in a truncated octahedron of water molecules obtained with the molecular mechanics force field Amber and for 150 ns. These simulation studies were also carried out with the two NOS enzyme isoforms in complex with the natural substrate and cofactors (NOS/heme/BH4/Arg) as a control, and to get an insight of the flexible regions of these enzymes and the key contacts of the natural substrates. By applying MD studies, in which both ligand and enzyme are considered flexible, 1) false positive binding modes in molecular docking can be discarded, as the ligand would be released from the selected pocket during simulation; 2) a more realistic conformation of the enzyme@ligand(s) complex is achieved since the intrinsic shape-changing motions of the enzyme are considered (induced-fit model); and 3) an insight of the stability and strength of the key ligand interactions can be obtained.

**FIGURE 6:**
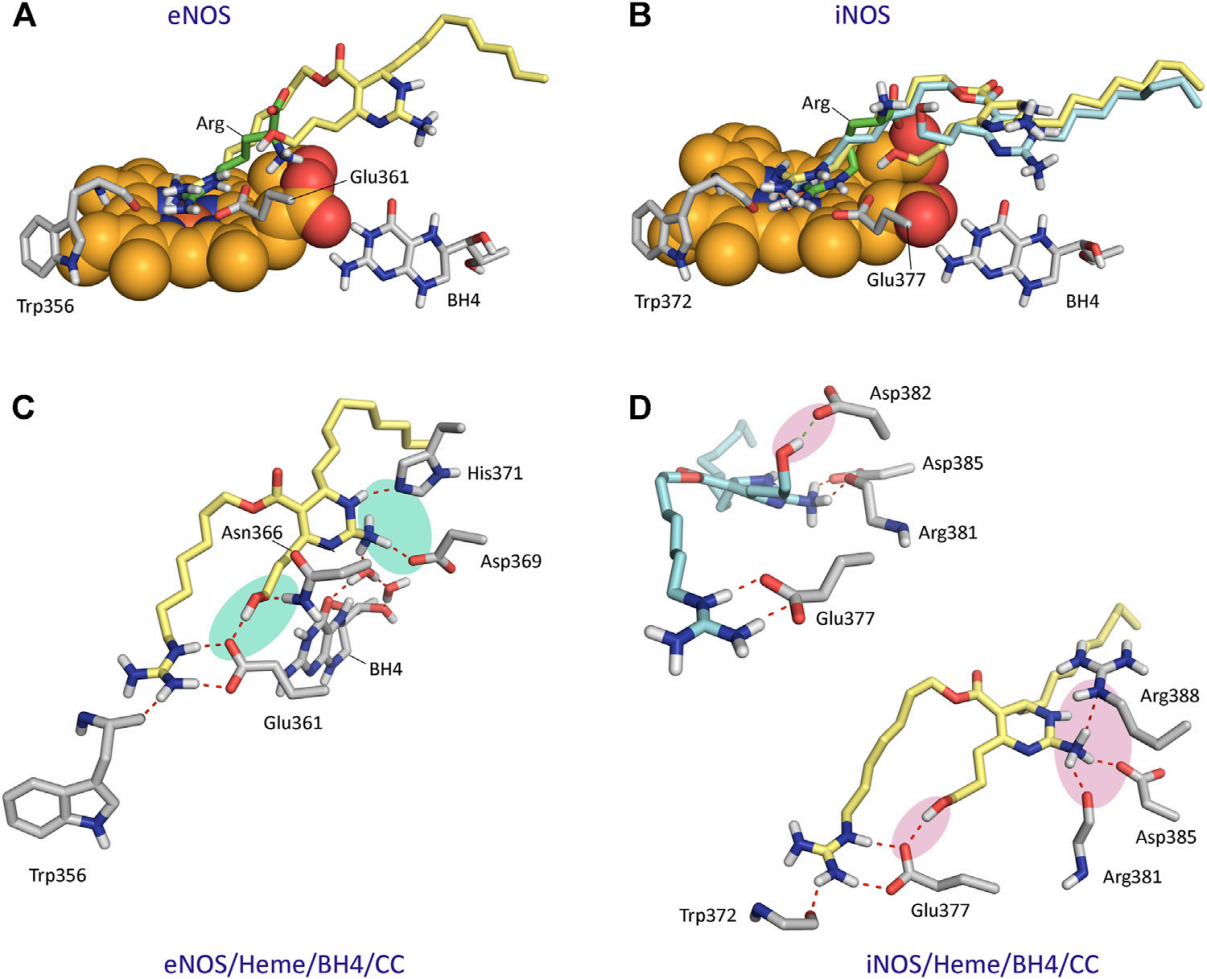
Binding mode of Crambescin C1 (CC) in the active site of NOS enzymes obtained by docking and MD simulation studies. **(A)** Comparison of the binding mode of CC (yellow) with the natural substrate (Arg, green) in the active site of eNOS. **(B)** Comparison of the two main poses of CC (yellow and blue) with the natural substrate in the active site of iNOS. The position of the heme group (spheres) and BH4 are shown **(A,B)**. **(C)** Interactions of CC with eNOS. Snapshot after 140 ns is shown. **(D)** Interactions of CC with iNOS. The major pose of CC is shown in yellow. Snapshots after 75 and 135 ns are shown. Relevant side chain residues are shown and labeled. Polar interactions between CC and the NOS enzymes are shown as red dashed lines. The most relevant polar interactions for panels **(C,D)** are highlighted with a shadow (blue and pink, respectively).

**FIGURE 7:**
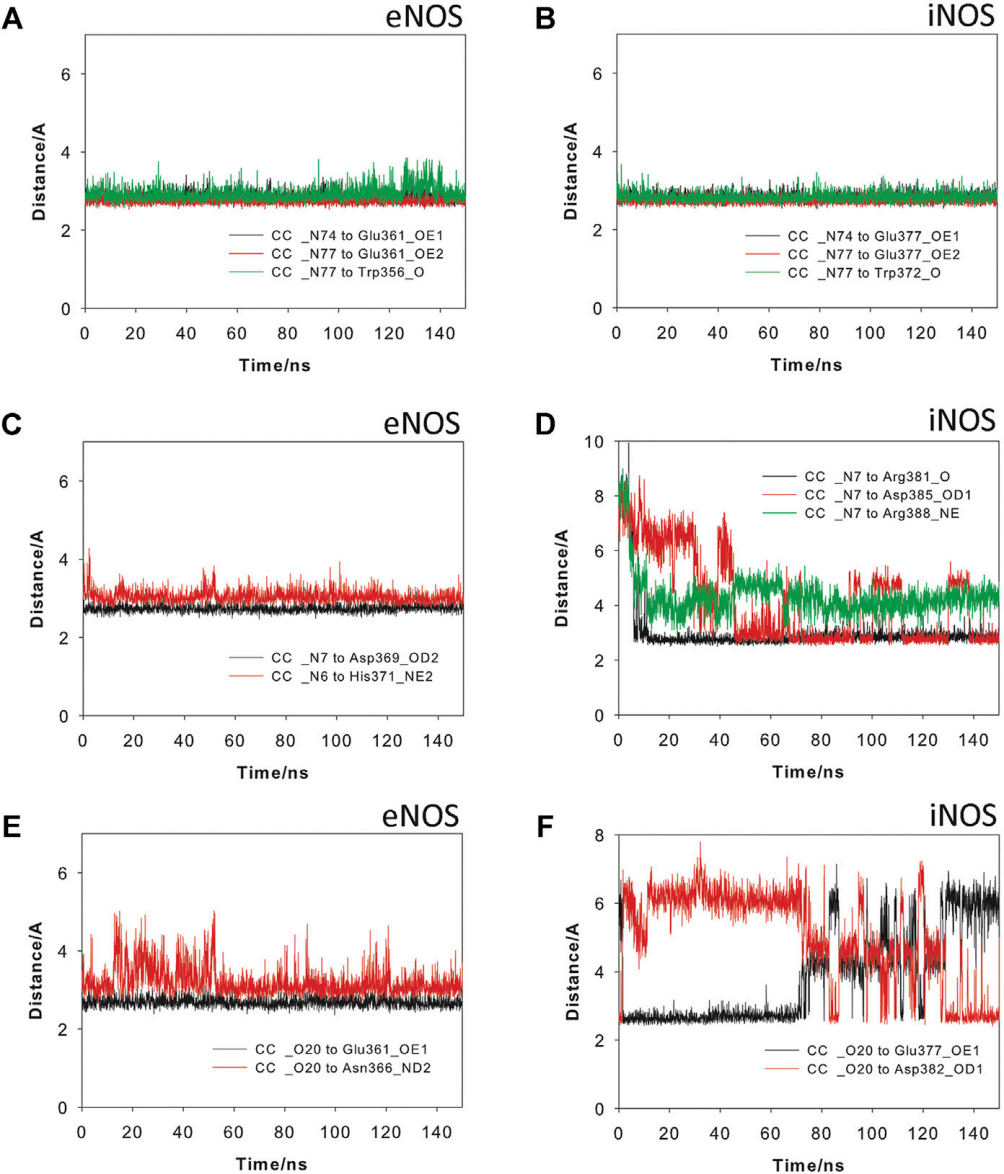
Variation of the most relevant electrostatic and hydrogen bonding interactions of Crambrescin C1 (CC) when binding to the eNOS **(A,C,E)** and iNOS **(B,D,F)** enzymes during the simulation (150 ns). Interactions involving the guanidinium **(A,B)** and pyrimidinium **(C,D)** moieties, and the hydroxylated side chain **(E,F)** are shown.

The results from 150 ns of dynamic simulation showed that the enzyme complexes proved to be stable during most of the simulation as revealed the analysis of the root-mean-square deviation (rmsd) of the protein backbone (Cα, C, N, and O atoms) in the NOS/Heme/BH4/L (L = Arg, CC or CA) complexes studies, which in all cases proved to be low (Supplementary Figure S2). As expected, in the first part (∼20 ns) of the simulation a small adjustment in the position of the ligands CC and CA and key side chain residues of the active site took place. Several conformations for the carbon side chain of the two ligands were identified during the simulation as expected for a long and flexible chain located in a wide pocket, which is the interface between the two enzyme chains (Supplementary Figure S3). But eventually, the arrangement of pyrimidine and guanidinium moieties of the two ligands, which proved to be responsible for the stronger binding interactions with the enzyme, revealed to be stable during most of the simulation (∼130 ns). However, our computational studies revealed significant differences in some of these relevant interactions of ligands CC and CA with eNOS and iNOS enzyme isoforms. CC would be anchored in the active site of the two NOS enzyme isoforms by mainly two key points: 1) the guanidinium group; and 2) the pyrimidine moiety (Figure 6). Non-polar interactions were also identified but since they appeared to be similar for the two ligands were not considered in this comparison. As for the natural substrate, the guanidinium moiety of CC would be anchored on top of the heme group by establishing a strong salt bridge with the carboxylate group of Glu361/Glu377 and by hydrogen bonding with the main carbonyl group of Trp356/Trp372 (in eNOS and iNOS, respectively). For eNOS, the NH_3_ group of the pyrimidine moiety in CC would establish an electrostatic interaction with Asp369 and the NH group would make a hydrogen bonding with the side chain of His371 (Figure 6C). In addition, indirect contacts of CC with BH4 *via* mainly the NH_3_ group and a water network would be also established. Remarkably, the position of the hydroxylated side chain in CC would be frozen in the eNOS active site by hydrogen bonding with the carboxylate group of Glu361 and the amide side chain of Asn366. It is important to highlight that the latter residues are involved in the natural substrate recognition. The strength and stability of these key contacts involving guanidinium and pyrimidine groups and hydroxylated side chain is clearly visualized by the analysis of the variation of the distances between the atoms involved in those interactions during the simulation (Figure 7). No significant changes were identified.

For iNOS, the guanidine of the pyrimidine moiety in CC would establish an electrostatic interaction with the carboxylate group of Asp385, as well as hydrogen bonding interactions with the guanidinium group of Arg388 and the carbonyl main chain of Arg381 (Figure 6). Remarkably, in contrast to eNOS, a more flexible arrangement of the hydroxylated side chain in CC was identified. Thus, our simulation studies revealed two main locations for this chain, involving in both arrangements strong hydrogen bonding interactions. Thus, the major pose would be similar to eNOS, thus including a hydrogen bond between the OH group and the carboxylate group of Glu377. For the minor arrangement, the OH group of the chain in CC would interact by hydrogen bonding with the carboxylate group of Asp382. The analysis of the variation of the distance between the atoms involved in the latter hydrogen bonding interactions during the whole simulation revealed that the contact with Glu377 would be almost three times more frequent than with Asp382 (Figure 7F). Besides the analysis of the amino acid sequence (Supplementary Figure S4) in the two NOS enzyme isoforms revealed that the active site is quite conserved, the replacement of an asparagine (Asn366) by an aspartate (Asp382) would be responsible of the higher flexibility of the hydroxylated side chain in CC when binding the iNOS enzyme. Moreover, the binding free energies of CC and the natural substrate were calculated using the MM/PBSA approach in explicit water (generalized Born, GB) as implemented in Amber (Table 1). The results revealed the higher affinity of CC than the endogenous substrate by the two NOS enzymes with calculated binding energy differences up to 30 kcal mol^−1^.

**TABLE 1:** Calculated Binding Free Energies using MM/PBSA^a^.

Enzyme	Ligand	Energy (kcal mol^−1^)	Relative energy difference (kcal mol^−1^)^b^
eNOS	Arg	−53.1 ± 0.2^c^	0
	CC	−76.5 ± 0.3^c^	−23.4
	CA	−59.3 ± 0.3^c^	−6.2
iNOS	Arg	−26.1 ± 0.2^c^	0
	CC	−58.9 ± 0.3^c^	−32.8
	CA	−49.1 ± 0.2^c^	−23.0

a Only the last 100 ns of the whole simulation were considered for the calculations.

b relative to the natural substrate (Arg) in the same active center.

c standard error of mean.

**FIGURE 8:**
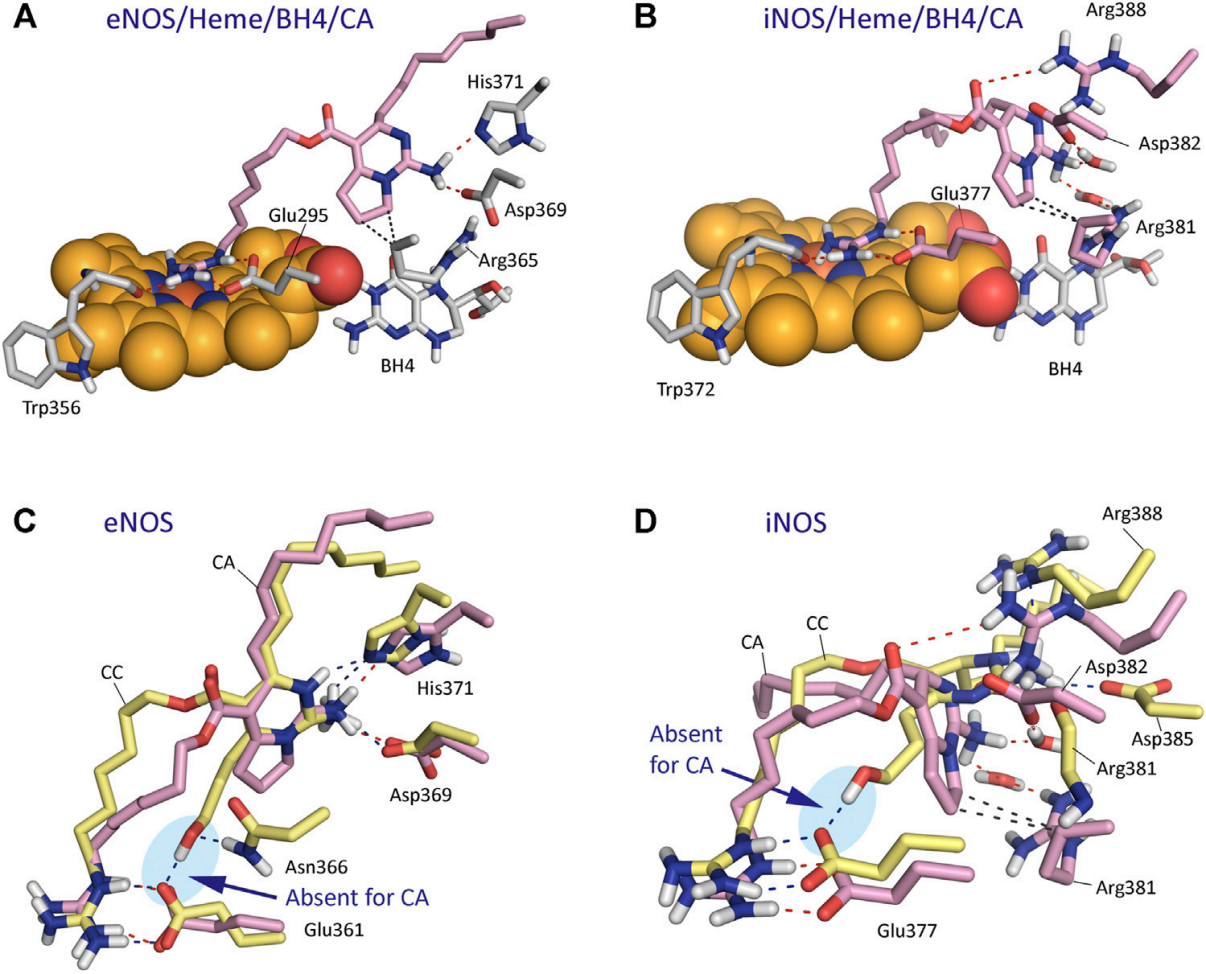
Binding mode of Crambrescin A1 (CA, pink) in the active site of NOS enzymes obtained by docking and MD simulation studies. **(A)** Overall view of the eNOS/Heme/BH4/CA enzyme complex. Snapshot after 140 ns of simulation is shown. **(B)** Overall view of the iNOS/Heme/BH4/CA enzyme complex. Snapshot after 80 ns of simulation is shown. **(C,D)** Comparison of the binding modes of CA (pink) and CC (yellow) with eNOS **(C)** and iNOS **(D)** enzymes. Relevant side chain residues are shown and labeled. The position of the heme group (spheres) and BH4 are shown (A,B). Polar (red, blue) and apolar (black) interactions between ligands and NOS enzymes are shown as dashed lines. Note how only CC would have an strong hydrogen bonding interaction with Glu361/Glu377 residues (blue shadow).

The results of our computational studies with CA and CC revealed that for eNOS both ligands have similar interactions involving the guanidinium and pyrimidine moieties (Figure 8). However, the replacement of the hydroxylated side chain in CC by a five-membered cycle would cause loss of one of the three main anchoring points of CC to the oxidase domain, specifically the residues Glu361 and Asn366 (Figure 8C). By this change, a significant loss of affinity of CA for the enzyme would be expected, as was also predicted by the calculated binding free energies (Table 1). Comparison of the binding modes of CA and CC with iNOS also revealed the lack of the hydrogen bond interaction with the conserved Glu377. In contrast to eNOS, significant changes were also identified involving the contacts of the pyrimidine moiety in CA. Specifically, the interactions of the NH3 group with the side chain residues of Asp382 and Arg381 were bridged by water molecules. Moreover, the guanidinium group of Arg388 would be interacting with the ester carbonyl group in CA.

### *In vivo* Effect of CC

**FIGURE 9:**
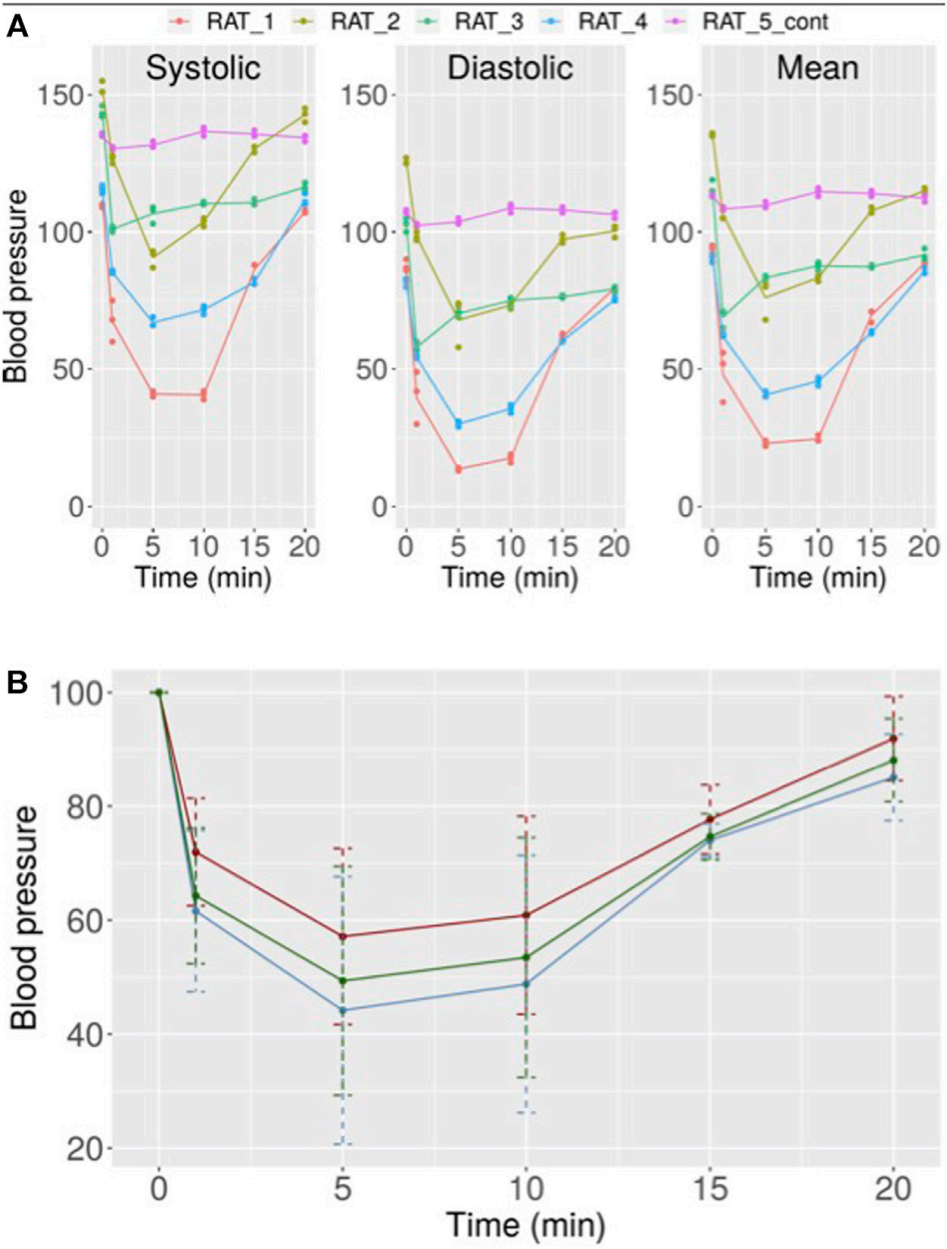
**(A)** Variation in systolic, diastolic, and mean blood pressure in rats after treatment with 10 µM CC. Independent results obtained for five rats are shown. **(B)** Mean values with error for the results obtained for all rats after treatment with 10 µM CC (red: systolic pressure, blue: diastolic pressure, green: mean pressure).

Finally, to assess NO production *in vivo* by CC, its effect on blood pressure was analyzed. The results showed that this compound induced a rapid, potent, and transient hypotension that lasted no more than 20 min. One minute after CC injection an important reduction in blood pressure was detected. This reduction peaked between 1 and 10 min and then a reversion was observed. At 15 min after CC injection, still a drop in blood pressure could be observed, while an almost complete recovery of normal blood pressure was observed after 20 min (Figure 9A). The means in percentage for the diastolic, systolic, and mean pressure are disclosed in Figure 9B.

## Discussion

### CC and HCC Are Potent Inducers of Metallothioneins

The gene expression analysis for HepG2 cells after treatment with the three crambescin analogues, CC, CA, and HCC, revealed significant differences between them in terms of number of genes altered and relative potencies, with CC showing the highest relative activity, followed by HCC and CA. The most striking effect of these compounds proved to be the induction of MTs that was clearly observed for the most structurally close compounds, CC and HCC, which only differ in one extra methylene group of the side chain containing the guanidinium group. This could be a consequence of several pathways induced by these compounds, including zinc exposure, induction of oxidative stress or increased NO production. Zinc has been previously shown to protect HepG2 cells against oxidative stress by MT induction (Perez and Cederbaum, 2003). Also, we previously observed MT induction without increased oxidative stress in CC treated HepG2 cells (Roel et al., 2015). After discarding the induction of oxidative stress as cause of the MT induction, and given the chemical structure of the crambescin analogues, we focused on NO production. Our studies with HepG2 cells in culture revealed an increased NO production after treatment with CC.

### CC and HCC Induce NO Production *in vitro.* Relaxation of Aorta Rings and *in vivo* Vasodilation

NO acts as a messenger molecule in the maintenance of vascular tone, neuronal signaling, and in the response to infections (Pfeiffer et al., 1999). In mammals the primary source of NO is nitric oxide synthase (NOS), which has three isoforms: neuronal NOS (nNOS), inducible NOS (iNOS) and endothelial NOS (eNOS). These enzymes catalyzed the oxidation of L-arginine to citrulline and NO in a two-step oxidation process. The first step is the *N*-hydroxylation of the guanidine group of L-arginine to give *N*-hydroxy-L-arginine, followed by the subsequent oxidation of this intermediate to give citrulline and NO (Stuehr et al., 1991).

In isolated rat aorta rings an important relaxation was observed in the presence of CC and HCC. In part the relaxation was endothelium dependent, higher in intact rings–with endothelium-than in rubbed rings–without endothelium-. Endothelial effects appear to be related to NO production by eNOS (constitutively expressed in the endothelial cells but not in the smooth muscle cells) given that the known eNOS inhibitor L-NA reverts the endothelial effect. CC and HCC also induce a significant higher relaxation in LPS pre-treated rubbed rings than in rubbed control rings. The higher effect in LPS pre-treated rings seems to be related to NO production by iNOS because LPS is an inducer of iNOS (Sirsjo et al., 1994) and the iNOS inhibitor 1400 W (Garvey et al., 1997) drastically reverts it. All together these results suggest that CC and HCC can modulate eNOS and iNOS, increase production of NO and relax vascular tone. Our experiment in rat aorta rings also rule out that the NO production by CC was specific for HepG2 cells. Bearing in mind that CC and HCC incorporate a guanidine group like L-arginine, the endogenous substrate of NOS, our studies also suggest that CC and HCC could be a good substrate of eNOS and iNOS enzymes and could facilitate their effect. While the relaxations elicited by CC and HCC at low concentrations appear to be mostly mediated by NOS, the mechanism responsible of NO-independent relaxations prominent at the highest drug concentrations tested, remain to be determined. The results obtained *in vivo* support the notion of NOS mediated rapid NO release after CC injection, based on the almost immediate vasodilation observed. A faster effect than that observed in humans after L-arginine infusion was observed (Bode-Böger et al., 1998).

In addition to L-arginine and some L-arginine analogues (Lee et al., 1999), it has been reported that some non *a*-amino acid *N*-hydroxyguanidines may act as NOS substrates (Garvey et al., 1997; Xian et al., 2002). That is the case of *N*-alkyl-*N*′-hydroxyguanidines, in which the alkyl substituent can be propyl, butyl, or pentyl, or some *N*-aryl-*N*′-hydroxyguanidines, in which those having electron-rich aryl groups showed to be the most active ones. Among them, *N*-butyl-*N*′-hydroxyguanidine is the best exogenous substrate of NOS reported, having a catalytic efficiency (*k*_cat_/K_m_) 2-fold lower than the endogenous substrate *N*-hydroxy-L-arginine, due to its 1.4-fold higher K_m_ and its 0.7-fold lower *k*_cat_. These compounds, which are mimetics of *N*-hydroxy-L-arginine, would act in the second step of the NOS catalyzed reaction in which *N* ^G^-hydroxy-L-arginine is converted into L-citrulline. The presence of the *a*-amino acid group in the endogenous substrate, or at least the strong interactions with the enzyme promoted by this moiety, seems to be essential for the first step of the NO biosynthesis, through which L-arginine is converted into *N* ^G^-hydroxy-L-arginine. This is supported by the fact that while simple *N*-substituted guanidine derivatives probed not to be enzyme substrates (Xian et al., 2002), conformationally restricted L-arginine derivatives (Lee et al., 1999), which contain an *a*-amino acid group and its guanidinium group is not hydroxylated, showed to be transformed by the enzyme. Our computational studies suggested that crambescin CC, which contains a pyrimidine moiety and a hydroxylated side chain in addition to the guanidinium group, would interact with the endogenous substrate binding pocket and could have higher binding affinity against iNOS and/or eNOS enzymes than L-arginine. Thus, the estimated binding energy differences relative to the endogenous substrate would be over 30 (iNOS) and 20 kcal mol^−1^ (eNOS), which can be directly related to a 2.6-fold (iNOS) and 1.4-fold (eNOS) increase on the dissociation constants of CC compared with L-arginine, as ΔG_binding_ = –2.303 RT pK. The higher affinity of CC would be mainly due to the additional strong hydrogen bonding interaction promoted by the hydroxylated side chain with the residues Glu377 and Asn366 (iNOS) and Glu361 and Asp382 (eNOS), which are involved in the *N*-terminal catalytic domain containing the heme active site. The affinity of CC by iNOS would be remarkably higher than for eNOS that seems to be due to: 1) differences in the amino acid sequence of the binding pocket of the cyclic moiety in CC, and, as a result, in the relative strength of the polar interactions (hydrogen bonding, and electrostatic); and 2) the existence of more direct contacts with the NOSs enzymes rather than interactions mediated by water molecules. Our computational studies also revealed that the replacement of the hydroxylated side chain in CC by an aliphatic five-membered cycle in CA would mainly cause the loss of aforementioned strong contact with the oxidase domain. In addition, the conformational restraint imposed by the bicyclic moiety in CA would promote differences in the anchoring of its guanidinium group on top of the heme group that would also contribute to decrease its affinity for the NOSs enzymes. Altogether, the results of our MD simulation studies suggested that the experimentally observed NO production might be due to an activation of the iNOS/eNOS enzymes by CC acting as an exogenous substrate. This hypothesis is supported by: 1) the results of the *in vitro* competitive studies using known iNOS (1400 W) and eNOS (L-NA) inhibitors and CC; 2) the molecular similarities between L-arginine and CC relating to their guanidinium groups and the interaction with the oxidase domain; and 3) as well as the great capacity of CC to anchor itself to the enzyme through a set of strong hydrogen bonding and electrostatic interactions via the pyrimidine and hydroxylated side chain moieties. Further kinetic experiments with isolated enzymes are required to corroborate this hypothesis.

The most recognized function of NO is the activation of the ubiquitously expressed soluble guanylate cyclase (sGC) which catalyses the conversion of GTP to the second messenger cGMP with a low basal rate. Expossure of sGC to NO (10–100 nM) induces an allosteric activation of the enzyme with a 500-fold increment in activity. The cGMP in turn regulates several kinases including protein kinase G (PKG), GPCRs, ion channels, phosphodiesterases, tyrosine kinases, and tyrosine phosphatases (Sirsjo et al., 1994). In the endothelium the NO derived from eNOS can cross cell membranes into the underlying vascular smooth muscle. In vascular smooth muscle PKG is the primary downstream target of cGMP. PKG activates the myosin light-chain phosphatase (MLCP) (Mansuy and Boucher, 2002) dephosphorylating smooth muscle myosin mediated by cGMP with the consequent abolishment of tonic contraction resulting in vasodilation (Gewaltig and Kojda, 2002). In this context, based on the results presented in this work, CC acts as substrate of eNOS rapidly liberating NO with the consequent rapid vasodilation. The increased vasodilation observed *in vitro* in the presence of endothelium when compared to rubbed vessels support this idea.

In conclusion, this work provides evidence of a new mechanism of action of several crambescin analogs involving the interaction of these molecules with eNOS and iNOS with rapid and transient release of NO *in vitro* producing aorta rings relaxation, and *in vivo* hypotension. The results from this study indicate that CC and other crambescins might be used as a guide to further develop new drugs useful to treat disease states in which the bioavailability of NO is perturbed.

## Data Availability

The original contributions presented in the study are included in the article/Supplementary Material, further inquiries can be directed to the corresponding authors.
