# Supplementary material for: Crambescin C1 Acts as A Possible Substrate of iNOS and eNOS Increasing Nitric Oxide Production and Inducing In Vivo Hypotensive Effect
Source: Front Pharmacol. 2021 Jul 7;12:694639. doi: 10.3389/fphar.2021.694639 (PMC8312399; doi:10.3389/fphar.2021.694639)
Supplement: Supplementary file 3 [file datasheet1.docx]

**Supporting Information for**

Crambescin C1 Acts as A Possible Substrate of iNOS and eNOS Increasing Nitric Oxide Production and Inducing In Vivo Hypotensive Effect

Juan A Rubiolo^1*^, Emilio Lence^2^, Concepción González-Bello^2^, María Roel^8^, José Gil-Longo^3^, Manuel Campos-Toimil^3^, Eva Ternon,^4^ Olivier P. Thomas^4,5^, Antonio González-Cantalapiedra^6^, Henar López-Alonso^6^, Mercedes Rodriguez Vieytes,^7^ Luis M Botana^8^*

Table of Contents

1. Figure S1..…………...…..………………..…………….……………...…….………….……………. S2

2. Figure S2..…………...…..………………..…………….……………...…….……………....………… S3

3. Figure S3.…………...…..…….…………..…………….……………...…….…………..……………. S4

4. Figure S4.…………...…..…….…………..…………….……………...…….…………..……………. S5

**Figure S1:** Representative charts showing: i) effects of acetylcholine (Ach) in intact and rubbed rings precontracted with phenylephrine (Phe); ii) effects of crambescin C1 (CC; panel A) and homocrambescin C1 (HCC; panel B) in intact and rubbed rat aortic rings precontracted with Phe and reversion of their effects in intact rings by N^G^-nitro-L-arginine (L-NA); iii) recovery of the Phe response after a washout period. The different concentrations are expressed as logarithms of molar concentrations.

**
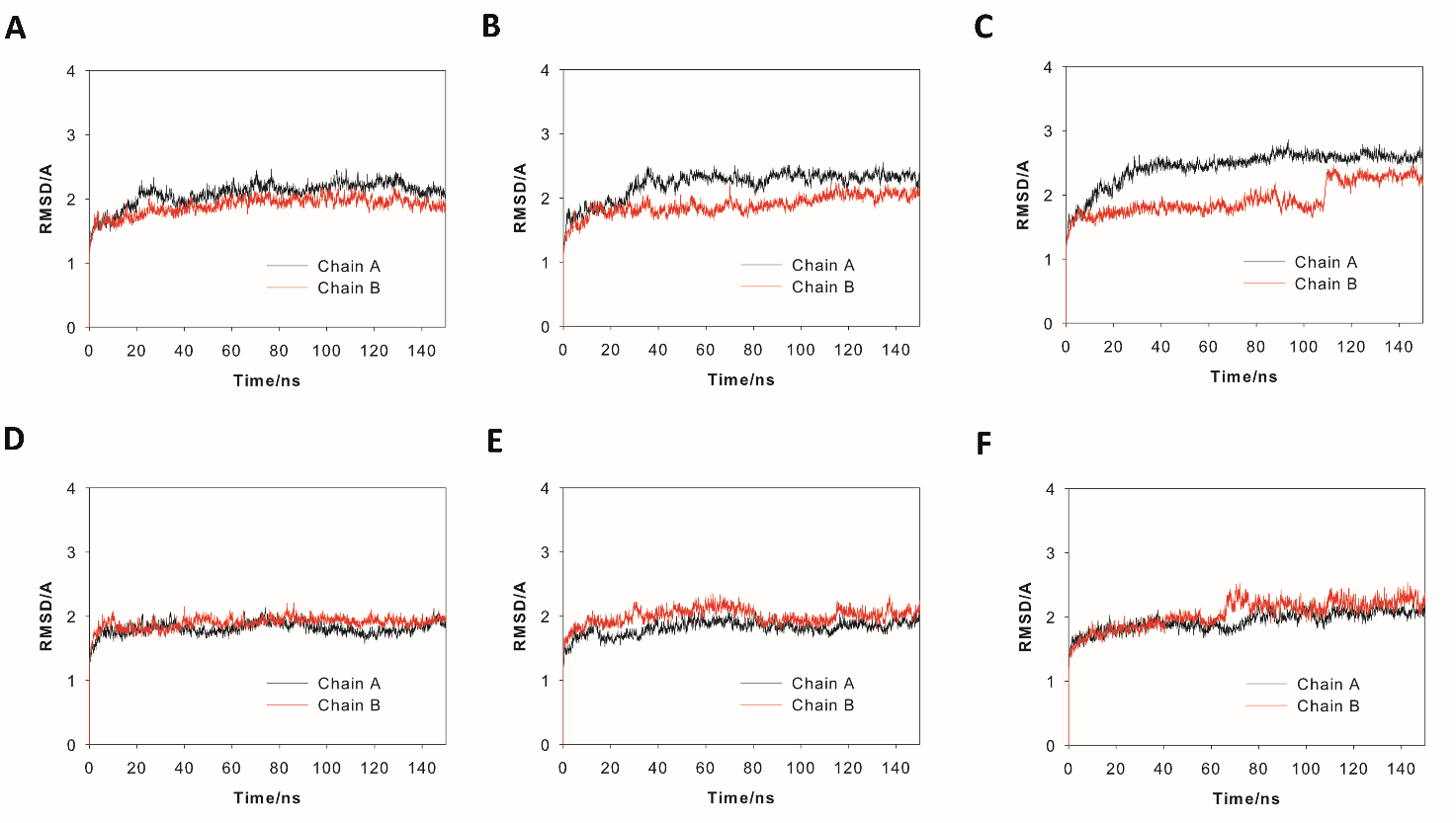
**

**Figure S2**: RMSD plots for the protein backbone (C^α^, C, N, and O atoms) calculated in the complexes obtained from MD simulations studies (150 ns): (A) eNOS/Heme/BH4/Arg; (B) eNOS/Heme/BH4/CC; (C) eNOS/Heme/BH4/CA; (D) iNOS/Heme/BH4/Arg; (E) iNOS/Heme/BH4/CC; (F) iNOS/Heme/BH4/CA. The results of the two chains are shown. Note how all complexes showed to be very stable during simulation as low variations on the RMSD values were obtained.

**
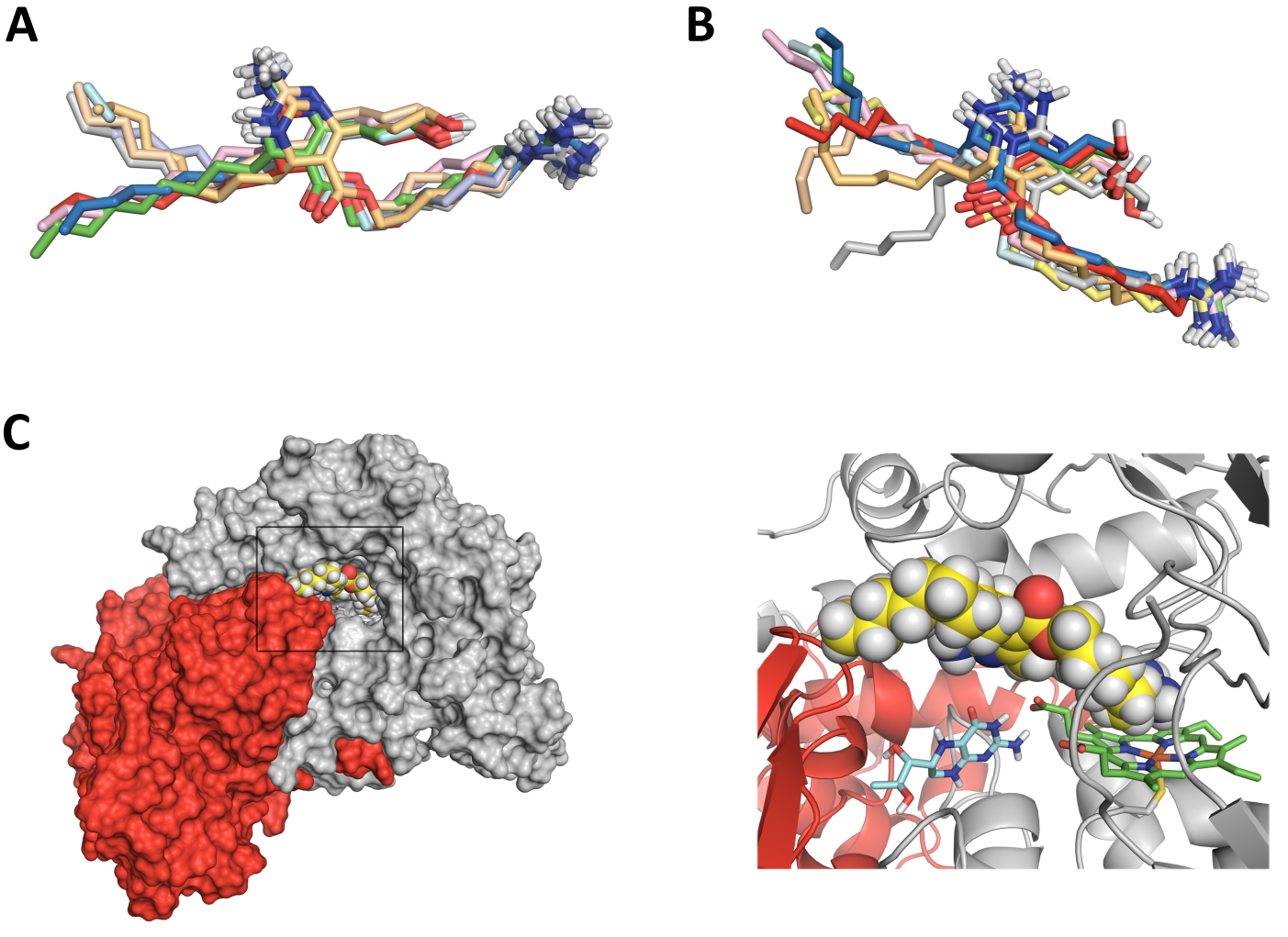
**

**Figure S3**: Comparison of several snapshots of CC in the enzyme complexes during simulations: (A) eNOS/Heme/BH4/CC (A) and (B) iNOS/Heme/BH4/CC. Note the high flexibility of the carbon side chain of the ligand, which is located in the interface between the two chains of the enzyme. (C) Overall view of the eNOS/Heme/BH4/CC enzyme complex. The long carbon side chain of Crambrescin C1, which is shown in spheres, is located in the dimmer interface. A close view is also provided. BH4 (cyan) and heme group (green) are shown in sticks. Chains A and B are shown in grey and red, respectively.

41 100

eNOS_4D1O APASLLPPAPEHSPPSSPLTQPPEGPKFPRVKNWEVGSITYDTLSAQAQQDGPCTPRRCL

iNOS_1NSI -----------------LDATPLSSPRHVRIKNWGSGMTFQDTLHHKAKGILTCRSKSCL

: * ..*:. *:*** * *** :*: * : **

101 160

eNOS_4D1O GSLVFPRKLQGRPSPGPPAPEQLLSQARDFINQYYSSIKRSGSQAHEQRLQEVEAEVAAT

iNOS_1NSI GSIMTPKSLTRGPRDKPTPPDELLPQAIEFVNQYYGSFKEAKIEEHLARVEAVTKEIETT

**:: *:.* * * *::** ** :*:****.*:*.: : * *:: * *: :*

161 220

eNOS_4D1O GTYQLRESELVFGAKQAWRNAPRCVGRIQWGKLQVFDARDCRSAQEMFTYICNHIKYATN

iNOS_1NSI GTYQLTGDELIFATKQAWRNAPRCIGRIQWSNLQVFDARSCSTAREMFEHICRHVRYSTN

***** .**:*.:**********:*****.:*******.* :*:*** :**.*::*:**

221 280

eNOS_4D1O RGNLRSAITVFPQRCPGRGDFRIWNSQLVRYAGYRQQDGSVRGDPANVEITELCIQHGWT

iNOS_1NSI NGNIRSAITVFPQRSDGKHDFRVWNAQLIRYAGYQMPDGSIRGDPANVEFTQLCIDLGWK

.**:**********. *: ***:**:**:*****: ***:********:*:***: **

281 340

eNOS_4D1O PGNGRFDVLPLLLQAPDEPPELFLLPPELVLEVPLEHPTLEWFAALGLRWYALPAVSNML

iNOS_1NSI PKYGRFDVVPLVLQANGRDPELFEIPPDLVLEVAMEHPKYEWFRELELKWYALPAVANML

* *****:**:*** .. **** :**:***** :***. *** * *:*******:***

341 400

eNOS_4D1O LEIGGLEFPAAPFSGWYMSTEIGTRNLCDPHRYNILEDVAVCMDLDTRTTSSLWKDKAAV

iNOS_1NSI LEVGGLEFPGCPFNGWYMGTEIGVRDFCDVQRYNILEEVGRRMGLETHKLASLWKDQAVV

**:******..**.****.****.*::** :******:*. *.*:*:. :*****:*.*

401 460

eNOS_4D1O EINVAVLHSYQLAKVTIVDHHAATASFMKHLENEQKARGGCPADWAWIVPPISGSLTPVF

iNOS_1NSI EINIAVLHSFQKQNVTIMDHHSAAESFMKYMQNEYRSRGGCPADWIWLVPPMSGSITPVF

***:*****:* :***:***:*: ****:::** ::******** *:***:***:****

461 488

eNOS_4D1O HQEMVNYFLSPAFRYQPDPW--------

iNOS_1NSI HQEMLNYVLSPFYYYQVEAWKTHVWQDE

****:**.*** : ** : *

**Figure S4:** Amino acid sequence alignments for crystal structures of eNOs (PDB code 4D1O, 1.82 Å) and iNOS (PDB code 1NSI, 2.55 Å)]. Protein sequences were aligned using the CLUSTAL Omega multiple sequence alignment (<http://www.ebi.ac.uk/Tools/msa/clustalo/>, accessed February 1, 2021). Identical residues in the proteins used in these studies are highlighted in blue.
